# RETRACTION: Involvement of Heme Oxygenase‐1 Participates in Anti‐Inflammatory and Analgesic Effects of Aqueous Extract of *Hibiscus taiwanensis*

**DOI:** 10.1155/ecam/9840453

**Published:** 2026-02-27

**Authors:** Evidence-Based Complementary and Alternative Medicine

**RETRACTION:** S.‐L. Liu, J.‐S. Deng, C.‐S. Chiu, et al., “Involvement of Heme Oxygenase‐1 Participates in Anti‐Inflammatory and Analgesic Effects of Aqueous Extract of *Hibiscus taiwanensis*,” *Evidence-Based Complementary and Alternative Medicine,* vol. 2012 (2012). https://doi.org/10.1155/2012/132859.

The above article, published online on 21 June 2012 in Wiley Online Library (https://wileyonlinelibrary.com), has been retracted by John Wiley & Sons Ltd.

The retraction has been agreed following an investigation of the concerns raised by *Actinopolyspora biskrensis* on PubPeer [1], which identified several concerns related to multiple figures in the paper. More specifically,

**Figure 5a:** The COX‐2 and B‐Actin bands are identical after a horizontal stretch.

**Figure 6a:** The image of the control and Carr panels shares overlapping features with Figure 8a of [2] and 4a of [3].

As a result of the investigation, the data and conclusions of this article are considered unreliable.

The authors have been informed of this decision but did not provide a response.
